# Concerns regarding complementary feeding practices among urban Chinese mothers: a focus group study in Xi’an

**DOI:** 10.1186/s41043-018-0151-3

**Published:** 2018-07-24

**Authors:** Xin Liu, Xia Liao, Qiannan Ren, Meng Luo, Lei Yang, Jing Lin, Jie Chang

**Affiliations:** 1 Department of Epidemiology and Health Statistics, School of Public Health, Xi’an Jiaotong University Health Science Center, 76 West Yanta Road, Xi’an, 710061 Shaanxi People’s Republic of China; 2 Department of Nutrition, The First Affiliated Hospital, Xi’an Jiaotong University Health Science Center, 227 West Yanta Road, Xi’an, Shaanxi People’s Republic of China; 3 The Second Affiliated Hospital, Xi’an Jiaotong University Health Science Center, 157 West 5 Road, Xi’an, Shaanxi People’s Republic of China; 4 Department of Child and Adolescent Health, School of Public Health, Xi’an Jiaotong University Health Science Center, 76 West Yanta Road, Xi’an, Shaanxi People’s Republic of China; 5 Department of Pharmacy Administration and Clinical Pharmacy, School of Pharmacy, Health Science Center, Xi’an Jiaotong University, 76 West Yanta Road, Xi’an, Shaanxi People’s Republic of China; 6 Center for Drug Safety and Policy Research, Xi’an Jiaotong University, 76 West Yanta Road, Xi’an, Shaanxi People’s Republic of China

**Keywords:** Complementary feeding, Food additives, Urban mothers

## Abstract

**Background:**

Complementary feeding (CF) is an important determinant of infant growth and development. However, CF practices are influenced by caregivers’ perceptions and knowledge. This study aimed to describe perceptions and factors that potentially influence CF practices among Chinese mothers living in Xi’an, a rapidly developing city in China.

**Methods:**

This focus group study included three discussion groups. Topics related to practices and concerns regarding CF were discussed among women with at least one child aged 4–36 months. A brief questionnaire was used to collect demographic information for mothers and their children.

**Results:**

Among study participants, the timing of starting CF for their children varied from age 4 to 8 months. Grain was ranked as the top food for CF, and homemade food was preferred to commercial CF products. Food additives and preservatives were the priority concerns when purchasing commercial baby food, particularly regarding uncertainty about their safety. In terms of nutrition, deficiencies in minerals and vitamins were of major concern. The issue of bio-availability of added nutrients in baby food was also raised during the discussions. Participants showed a strong reliance on information obtained from the Internet via computers or smartphones as their main source of CF knowledge, but felt this information lacked expertise.

**Conclusions:**

Participating mothers from Xi’an prefer homemade food for CF to commercial products. More scientific knowledge of CF and related food safety issues should be available, perhaps via Internet-based approaches.

## Background

Human breast milk is universally regarded as the optimal nutrition source for term newborns [1]. However, for infants aged 6 months or older, breast milk alone may not meet nutritional and energy requirements for their rapid growth and development [2]. The World Health Organization’s guiding principles for complementary feeding (CF) recommend that additional foods with high nutrition qualities should be provided to children aged 6 months or older, regardless of whether or not they are breastfed [3]. In some low-income countries, inappropriate CF (e.g., inadequate in terms of vegetables, fruits, animal products, or iron-rich food) has been linked with stunting or anemia [2, 4]. Over the last decade, Ying Yang Bao, a soybean powder-based complementary food supplement incorporating micronutrients, was developed to improve the nutritional status of young children aged 6–24 months in China and promoted nationally [5]. To date, most studies on CF in China have focused on rural areas, generally in the middle and west of the country [6, 7], where the economy tends to lag [4, 8, 9].

A nutrition survey conducted among eight cities in China reported excessive intakes of vitamin A and zinc among infants [10]. The diversity and amount of food products have exploded in China as a result of rapid urbanization and economic development during the last 30 years, along with increased information and misinformation regarding nutrition and health [11]. Currently, the predatory marketing practices of multinational food companies across the world have substantially contributed an overnutrition status worldwide [12]. As estimated by the research firm Analysys, China’s market value for products for mothers and babies was 244 billion US dollars in 2015 and is forecasted to double by 2020 [13]. The Maternal Infant Nutrition Growth study conducted in eight cities across China reported urban infants aged over 6 months were characterized by high consumption of traditional grains (rice, noodles, millet, and steam bread), and around half did not consume any fruits or vegetables [14], which had been shown to be associated with stunt or anemia [2]. In recent years, various food safety events have occurred in China, which decreased public trust in commercial food choices [11]. With the existing developmental inequality among rural and urban areas of China, CF practices face risks in terms of nutrition deficiency and overnutrition. Similar situations have also been observed in India [15].

It is the parents who decide the time, content, and manner of introducing CF to their babies. This decision making is critical for their feeding actions. However, for urban Chinese mothers, little evidence is available that addresses the major concerns regarding CF, particularly as plenty of products, information, and choices are available and public trust in food safety was not satisfying. Therefore, we conducted a focus group study among mothers living in Xi’an, a rapidly developing city in China, to assess their perceptions and factors that potentially influenced their CF practice.

## Methods

### Participants

This focus group study used a qualitative approach to collect information on participants’ perceptions, attitudes, and other subjective information on a given topic [16, 17]. Eligible participants were mothers aged 18–45 years living in Xi’an, the capital city of Shaanxi Province, with at least one child aged 4–36 months. Exclusion criteria were as follows: (1) having a hearing impairment or difficulties in speaking or understanding Chinese, which may obstruct communication during the discussion; (2) current use of antidepressants; (3) with a child with birth defects including Down’s syndrome, congenital heart disease, neural tube defects, cleft palate, and hydrocephalus; and (4) with a child needing long-term drug treatment (> 3 months). These criteria were set in an effort to (1) ensure the capability of understanding and answering the questions, (2) collect as much information as possible about the topic, and (3) collect information on general infants rather than special patients. Participants were enrolled through the WeChat platform. A WeChat advertisement and quick response code was posted outside Lijiacun Wanda Center, a typical shopping and traffic center in Xi’an. By scanning the quick response code using a smartphone, potential participants could read the full study introduction and obtain contact information for the researchers. In China, WeChat is the most popular smartphone application used to receive and send messages, news, and other information, and has been used in both clinical and research practices [18, 19]. All researchers and field workers were trained before participating in this study. The study protocol was approved by the Ethical Committee of Xi’an Jiaotong University Health Science Center. Written informed consent was obtained from all participants.

### Procedures

Three focus group discussions were conducted at a meeting room in the Lijiacun Wanda Center for Maternal and Infant Care in Beilin District between December 2015 and January 2016. In total, 22 women participated in our study. One participant was excluded from the analysis because she did not complete the discussion. Data for 21 participants were included in the present analysis. There were 5–8 participants in each focus group discussion. Before the discussion, participants completed a questionnaire that collected information on age, race, education attainment, and household income level, as well as her child’s age, sex, birth weight, delivery mode, and age at starting CF. Participants also self-reported their height and weight, and their body mass index was calculated. Based on a focus-group guide [20], the discussions were semi-structured and organized by sequentially introducing topic questions that covered the following: general practice of CF, influencing factors, and approaches used to access knowledge about CF. Six topics were developed by referring to related local studies [8, 21] and an internal discussion: (1) Please list the food you added to your baby’s meal, besides breast milk or formula milk. Are they homemade or commercial products? (2) Are there any difficulties in preparing complementary food at home? Please describe them. (3) Please list factors that you would consider when buying complementary baby food. (4) Which nutrients would you pay attention to when selecting baby food? (5) What health beneficial effects or health claims were you interested in when selecting baby food? (6) What approaches did you use to get knowledge of complementary feeding?

Each discussion was moderated by the leading researcher (XL), with help from two assistant researchers (ML, LY). All discussions were audio recorded. First, the moderator explained the concept of CF and procedure of the discussion. Then, the topic questions were sequentially initiated by the moderator. All participants were encouraged to express their views in as much detail as possible for each topic and to express any different views. During the discussion, the moderator would ask for clarification and offer prompts to inspire extension if unexpected topics emerged. After all participants had made their statements, the moderator asked whether there were any further viewpoints to be added. Each focus group discussion lasted around 60 min.

### Data analysis

For the quantitative analysis, characteristics of participating women or children were presented as mean ± standard deviation for continuous variables, or number (%) for categorical variables. The quantitative analysis was carried out by using SPSS 13.0 for Windows (SPSS Inc., Chicago, Illinois, USA). Content analysis: All focus group discussions were recorded in Chinese. The audiotapes were then transcribed verbatim, and the transcripts translated into English. Two coders (XL, QR) independently reviewed the transcripts. After carefully reading the transcripts multiple times, the coders labeled related words or phrases in an effort to explore potential themes [22]. Then, a coding book was developed using those labels to record emerging themes [20]. Differences between two coders were discussed with a coauthor with experience in conducting qualitative studies (JC) and resolved by consulting another experienced researcher who had successfully conducted a focus group study on the acceptability of brown rice [23]. Several themes were identified in these discussions. The qualitative results were summarized or presented textually to demonstrate the findings.

## Results

### Participants’ characteristics

The mean age of participants was 31.6 ± 3.0 years, and their average body mass index was 20.4 ± 2.5 kg/m^2^. The majority of participants (76.2%) had attended college (Table 1), and 80% reported a monthly household income above 6000 Yuan (~ 950 USD) (Table 1). The age of participants’ children ranged from 10 to 35 months, and their birth weight ranged from 2.2 to 4.6 kg. The time of starting CF varied from 4 to 8 months, with the majority (71.4%) having started CF when their children were aged 6 months (Table 2).

**Table 1:** Characteristics of participating mothers (*N* = 21)

Characteristics	Mean ± SD or *n* (%)
Age (years)	31.6 ± 3.0
Weight (kg)	54.2 ± 6.0
Body mass index (kg/m^2^)	20.4 ± 2.5
Education attainment	
10–12 years	5 (23.8)
> 12 years	16 (76.2)
Monthly household income^a^	
3000–6000 RMB (~ 475–950 US$)	4 (20.0)
6001–10,000 RMB (~ 950–1600 US$)	13 (65.0)
> 10,000 RMB (~ > 1600 US$)	3 (15.0)
Parity	
1	19 (90.5)
2	2 (9.5)

*SD* standard deviation

^a^One participant did not provide information

**Table 2:** Characteristics of the children (*N* = 21)

Characteristics	Mean ± SD or *n* (%)
Age (months)	20.1 ± 7.1
Birth weight (kg)^a^	3.3 ± 0.5
Male	9 (42.9)
Delivery mode	
Eutocia	13 (61.9)
Cesarean section	8 (38.1)
Age of starting supplementary feeding	
4 months	1 (4.8)
5 months	3 (14.3)
6 months	15 (71.4)
8 months	2 (9.5)

*SD* standard deviation

^a^Two participants did not provide information

### CF practices and difficulties

All participants agreed with the importance of CF, and listed the food categories that they added to their children’s diets. Grain was ranked as the top food for CF, followed by eggs, vegetables, meat, and fruits (Table 3). Most mothers preferred to prepare baby food at home, but a few reported buying commercial products (Table 4).

**Table 3:** Complementary food items and frequency mentioned by participants

Food item	Number of participants mentioned
Grain	19
Eggs	17
Vegetable	13
Meat	9
Fruit	8
Cakes	1
Biscuits	1

**Table 4:** Representative responses of participants

Topics	Responses
Feeding practice	1. “I usually prepared food for my child by myself, and tried to add some commercial rice flours a few times, but my child did not like it. I used to boil hand-made noodles, or commercial children noodles, with additional blending of vegetables or meat mince.” 2. “We fed our baby with porridges, steamed eggs, and noodles. In very few cases, we bought baby food. For commercial products, we only added rice flour and biscuits.” 3. “We began to add rice flour when our baby aged 6 months old, and bought him various baby noodles.” 4. “I gradually added yolk, rice flour, fruit puree, and vegetable juice to our baby after 6 months old, with no much difficulty. For fruits and vegetable, we just chopped with knife, and occasionally used food processer.” 5. “I am a full-time mother, and I have no difficulty in preparing baby food.”
Difficulties in CF	1. “The time for preparing food for my child is limited, because we cook for him separately.” 2. “My child used to be very picky.” 3. “Sometimes, my child was attracted by TV program and could not keep eating at the table.”
Influencing factors in complementary feeding	1. “Some of the commercial products were added with many additives to make them attractive to babies. We believe the added stuff harmful.” 2. “The additives in baby food were invisible, so we have no idea how much they have added. I usually cook for my baby by myself.” 3. “I would consider whether the products contain additives and preservatives, when buying rice flours for my child.” 4. “I usually chose those well-known brands and organic products, by considering baby’s personal taste.” 5. One participant strongly stated, “All the food purchased for our baby is imported. We do not consider domestic food at all.” Another participant noted: “The rice flour we bought for our baby are mostly imported.”
Nutrition and health concerns	1. “When having regular physical examination, the general practitioner told us our child calcium deficiency. After that we paid more attention to calcium and added calcium supplement.” 2. One said: “I have no idea about the bioavailability of those added nutrients, given the current amount in the products.” 3. Another mother noted that: “I do not know whether there is an effect of those claimed stuff added in the food.” 4. “I am not sure how much my baby could absorb or digest those claimed nutrients.”
Approaches in getting knowledge of CF	1. “I usually get complementary food information from internet, smart phone, and WeChat. I generally trust those information, and it’s better to have more professional guidance.” 2. One participant said “Face to face lecture given by paediatricians or puericulturists is a good way of getting feeding information, because we could ask specific questions.” 3. One participant noted that “The knowledge should be well structured, systematic, and individualized, because the feeding practices differ by age and person.”

During the discussions, several participants reported difficulties in cooking for babies, and some reported they lacked time to prepare baby food in addition to meals for other family members. Some participants reported picky eating, difficulties in focusing on eating, or even food refusal among their children. During the discussions, participants also mentioned some solutions to these problems, including fixing the time for each meal, changing tastes or cooking styles, rewarding children using toys or smartphone games, and forcing the child to eat.

### Factors influencing CF

**Fig. 1:**
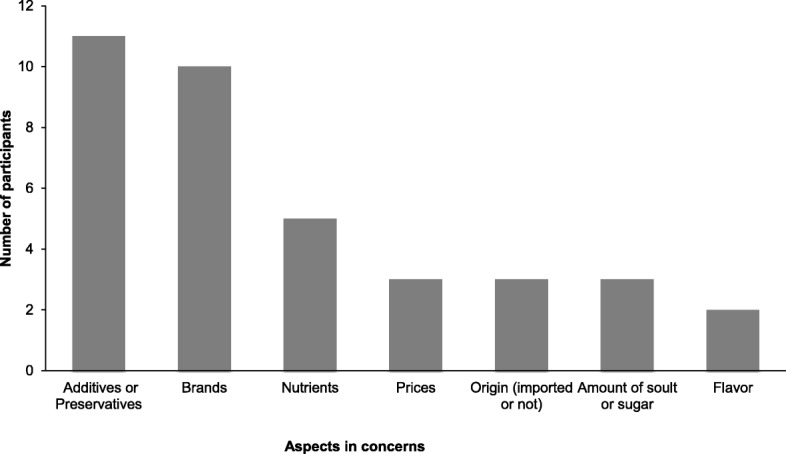
Number of participants who mentioned factors of concern in buying complementary foods

For most participants, priority concerns when purchasing complementary food products were additives or preservatives in the products (Fig. 1). Brands, prices, and nutrition were also frequently mentioned. Other participants emphasized the product origin and preferred imported products (Table 4).

### Nutrition and health concerns

When discussing nutritional issues with CF, about half of the participants highlighted concerns regarding deficiencies in minerals (*n* = 12) and vitamins (*n* = 10). Calcium, iron, and zinc were frequently listed as of major interest when checking food ingredients. The necessity of additional fortification of fatty acids in rice flour was discussed, particularly for docosahexaenoic acid (DHA) and eicosapentaenoic acid (EPA). Some participants thought it was necessary to add some nutrients. For example, EPA and DHA are mainly from deep-sea fish, which may be difficult to obtain in the daily diets of people living inland. However, some mothers argued that the unique smell of fish oils were not easily accepted by their babies. Some participants also expressed uncertainty about the real effects of added nutrients (Table 4).

Participants noted that all nutrients should be comprehensively balanced, rather than emphasizing individual nutrients. Regarding health effects in CF, some participants mentioned mental development and gastrointestinal comfort, while others noted that beneficial effects (e.g., for physical growth, immunity, and vision) were important in making CF decisions.

### Approaches in obtaining knowledge about CF

Eighteen of the 21 participants reported they usually obtained information about CF via the Internet. Baidu, the largest search engine in China, was a commonly used tool, with WeChat, “Sina Weibo,” and other smartphone applications also noted as information sources. Some participants also relied on CF information from books (*n* = 9) and friends, parents, or relatives (*n* = 7) when having difficulties. A few participants believed that suggestions from pediatricians and puericulturists were more reliable than Internet information (*n* = 3). Instructions provided on commercial food products were reported as a way to gather information by one participant. Based on their experiences, some participants made suggestions for improvement of accessibility of CF information (Table 4).

## Discussion and conclusions

In this qualitative study, we conducted three focus-group discussions on CF practices among mothers living in a rapidly developing city in China. The majority of mothers preferred to prepare baby food at home, despite the continuing increase in commercialization of complementary food products. Food additives, brands, and nutrients were the three most frequently mentioned concerns when purchasing commercial baby food. Furthermore, participants exhibited a strong dependency on the Internet or smartphone applications for obtaining CF knowledge, but tended to feel this information lacked expertise.

To our knowledge, this is the first focus group study addressing the practices and factors that potentially influence CF among Chinese urban mothers. A focus group study is particularly useful in obtaining opinions in a social context where others’ views are freely discussed [16]. Decision-making for CF may be influenced by family members, friends, and advertisements from mass media. Using a focus-group discussion approach, Nielsen et al. identified different parental concerns relating to CF for children aged 7 and 13 months [24]. The Chinese population has undergone rapid social economic development and dramatic nutrition transition over the past few decades [25]. With the development of e-business and the increasing frequency of international travel, various processed food products have been introduced or directly imported to China [26]. One of the most frequently discussed categories is baby food. Unlike formula milk, which has been well-studied [27], complementary food has received less attention. Yue and colleagues conducted a qualitative study among caregivers in poor rural regions in China and reported a lack of basic knowledge about infant nutrition, despite poverty making no contribution to this situation [28]. There are large urban-rural discrepancies in China, and it is not clear to what extent urban caregivers could adapt to unfamiliar “food,” given their long-term homemade feeding patterns [8]. Our findings provide valuable information that may be helpful for local public health workers in urban areas to obtain an overview of mothers’ attitudes towards CF.

When discussing the acceptability of commercial baby food, participants were cautious about food additives or preservatives. Similarly, in a survey conducted among 430 consumers in Soul, Korea, 76.7% of participants thought that the food additives approved by the government were not safe [29]. In China, public trust in food safety is low. An important reason for this could be the illegal blending of substances in commercial food; for example, Sudan IV added to poultry feed to stain the egg yolk, melamine mixed into formula milk, and the use of ractopamine in pork [13, 30]. These events not only caused serious public health hazards but also led to loss of public trust in the food regulatory systems [31] and have increased food scares. According to a study conducted in Soochow, China, public risk perceptions of food safety were significantly mediated by concerns about additive safety [32]. This could be the major reason why an increasing proportion of Chinese mothers prefer imported baby food, as noted by our participants. The National Health and Family Planning Commission of China renewed the standard of uses of food additives in 2015, in which additives for baby food are strictly limited by category and amount. Moreover, the government has also committed to taking action to address food safety problems by strengthening monitoring, surveillance, and legislation [30]. On June 30, 2017, the General Office of the State Council of China launched the National Nutrition Plan 2017–2030 [33]. This provides national guidelines for governmental and commercial activities in nutrition development in China for the coming decade. The plan has specific emphasis on CF improvement for early life 1000-day nutrition action and has specified aims in food safety legislation and knowledge transmission. In addition, governmental regulations may also play a role in limiting health claims on the commercial products in the future. To build public trust in commercial baby food, we believe that knowledge about food safety-related issues (e.g., food additives) may need to be clearly delivered to consumers, instead of relying on “no additives” labeling or avoiding the issue.

Another potential health risk associated with commercial CF may be added sugar, which is linked to childhood obesity [34]. In particular, the total daily amount of sugar or salt should be given more attention when applying different food compositions. Homemade baby food approaches reported in our study were simple, and our participants believed they were good at making homemade food. However, homemade food could be improved by altering the cooking methods and increasing the variety of ingredients [35, 36]. If prepared appropriately, homemade foods could be a safe CF choice.

Our participants showed strong reliance on the Internet to obtain CF knowledge. However, they expressed uncertainties about information obtained from the Internet, and indicated a strong desire for expertise from doctors. This may call for new approaches or programs to be developed to balance these two demands, and enable convenient access to sufficient evidence-based guidelines or suggestions. Similar to the World Health Organization recommendations [3], most of our participants (71.4%) initiated CF when their child was aged 6 months, although some reported slightly earlier or later initiation. Timely introduction of CF with adequate nutrients is important, as breast milk after 6 months postpartum may no longer meet infants’ requirements. However, a systematic review linked early initiation of CF (3 months, 4 months, or 20 weeks) with higher risk for obesity during childhood [37]. In China, further education programs may be needed to introduce appropriate CF practices to the public, including information on the timing of CF.

We collected subjective attitudes or opinions; therefore, our recall-based approaches were less likely to introduce bias than quantitative studies. However, the self-reported values (including weight and height) might have had limited validity. With high education levels, our participants might have had higher expectations of CF information and more knowledge than those with a lower educational attainment. The number of participants in our study was also limited, and we cannot exclude the possibility that our data lacked saturation or central tendency given the sampling approach used. Therefore, the generalizability of our findings may be limited. More qualitative and quantitative studies are needed to further investigate the factors influencing CF among families of different socioeconomic status.

This study identified that food additives were the priority concern when choosing commercial CF, and most mothers’ concerns related to nutritional deficiencies. Moreover, further integration of CF information in terms of advanced routes of information transmission and expertise in infant nutrition knowledge are needed.

## Abbreviations

CF: Complementary feeding
DHA: Docosahexaenoic acid
EPA: Eicosapentaenoic acid
